# Steroidal and pregnane glycosides from *Ypsilandra thibetica*

**DOI:** 10.1007/s13659-011-0039-z

**Published:** 2012-01-14

**Authors:** Hai-Yang Liu, Chang-Xiang Chen, Yi Lu, Jun-Yun Yang, Wei Ni

**Affiliations:** State Key Laboratory of Phytochemistry and Plant Resources in West China, Kunming Institute of Botany, Chinese Academy of Sciences, Kunming, 650201 China

**Keywords:** *Ypsilandra thibetica*, Liliaceae, furostanol glycoside, pregnane glycoside, ypsilandroside

## Abstract

The whole plants of *Ypsilandra thibetica* have been analyzed as part of a systematic study on saponin constituents of medicinal plants. This has resulted in the isolation of two new bisdesmosidic furostanol saponins, named ypsilandroside P (**1**) and ypsilandroside Q (**2**), and one new pregnane glycoside, named ypsilandroside R (**3**), together with nine known steroidal glycosides. Their structures were elucidated on the basis of extensive spectroscopic analysis, including that of 2D NMR data, and the results of acidic hydrolysis. Ypsilandroside P (**1**) was cytotoxicity against two human tumor cell lines.

**Electronic Supplementary Material:**

Supplementary material is available for this article at 10.1007/s13659-011-0039-z and is accessible for authorized users.

## Introduction

In a continuation of our study on saponin constituents of medicinal plants, we have examined the saponin riched fraction prepared from the EtOH extract of the air-dried whole plants of *Ypsilandra thibetica* (Liliaceae). This perennial plant distributes in southwestern China and has been used as hemostatic agent in Chinese folk medicine.[1,2] In our recent study, we found that this species was a rich source of steroidal saponins. Two sapogenin, 22 spirostanol saponins, and two C-22 steroidal lactone glycosides were obtained from the title plants.[3–6] Further phytochemical investigation has been carried out on this species, with particular attention to the steroidal glycoside constituents, and has resulted in the isolation of two new bisdesmosidic furostanol saponins (**1** and **2**) and one new pregnane glycoside (**3**), together with nine known steroidal glycosides: protoprogenin II (**4**),[7] proto-Pb (**5**),[8] saponin Th (**6**),[9] pseudoproto Pb (**7**),[10] pregnane 5,16-dien-3*β*-ol-20-oxo 3-*O*-*α*-L-rhamnopyranosyl-(1→4)-*α*-L-rhamnopyranosyl-(1→4)-[*α*-L-rhamnopyranosyl-(1→2)]-*β*-D-glucopyranoside (**8**),[11] smilaxchinoside B (**9**),[12] parispseudoside C (**10**),[13] parispseudoside A (**11**),[13] and 26-*O*-*β*-D-glucopyranosyl-3*β*,26-dihydroxy-20,22-*seco*-25(*R*)-furost-5-en-20,22-dione 3-*O*-*α*-L-rhamnopyranosyl-(1→4)-*α*-L-rhamnopyranosyl-(1→4)-[*α*-L-rhamnopyranosyl-(1→2)]-*β*-D-glucopyranoside (**12**).[14] This paper reports the isolation, structural determination, and cytotoxic activity of these glycosides.

## Results and Discussion

Compound **1**, obtained as a white amorphous powder, gave a pseudo-molecular ion peak $[M-H]^-$ at *m/z* 1207.5736 (calcd. 1207.5747) in its HRESIMS. Combined with $^{13}C$ NMR spectroscopic data (Table 2), its molecular formula was

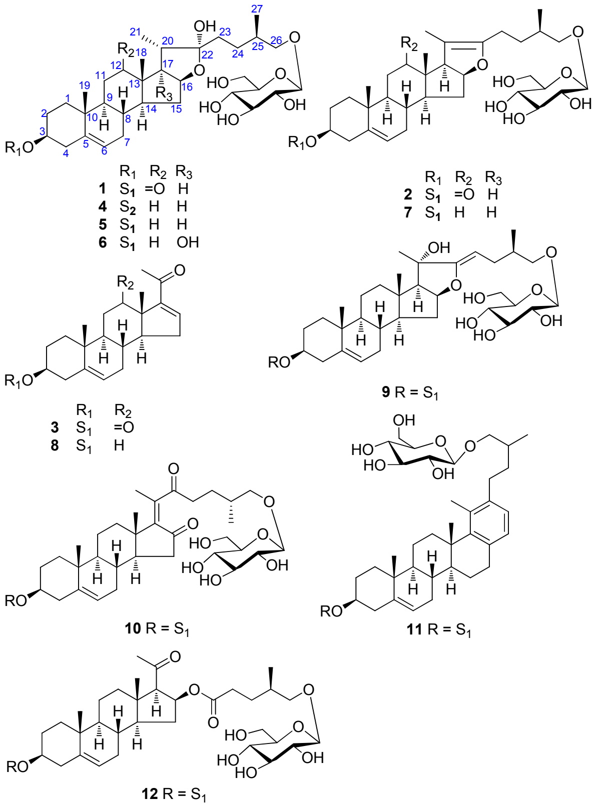

$S_1$ = *α*-L-Rha (1→2)-[*α*-L-Rha (1→4)-*α*-L-Rha(1→4)]-*β*-D-Glc-
$S_2$ = *α*-L-Rha (1→4)-*β*–D-Glc-

*To whom correspondence should be addressed. E-mail: haiyangliu@mail.kib.ac.cn

**Table 1.** $^{1}$**H NMR spectral data for compounds 1–3 (*δ* in ppm, *J* in Hz, $C_5D_5N$)**[a]

| Pos. | **1**[b] | **2**[c] | **3**[c] | Pos. | **1**[b] | **2**[c] | **3**[c] |
|---|---|---|---|---|---|---|---|
| 1a | 1.96, m | 1.53, m | 1.47, m | Glc-1′ | 4.93, d (7.2) | 4.91, d (7.9) | 4.93, d (7.4) |
| 1b | 0.89, m | 0.89, m | 0.87, m | 2′ | 4.18, m | 4.20, m | 4.21, m |
| 2a | 2.00, m | 2.00, m | 1.63, m | 3′ | 4.20, m | 4.23, m | 4.20, m |
| 2b | 1.78, m | 1.88, m | 1.41, m | 4′ | 4.39, m | 4.41, m | 4.40, m |
| 3 | 3.81, m | 3.82, m | 3.81, m | 5′ | 3.60, br. d (9.1) | 3.61, m | 3.59, br. s |
| 4a | 2.80, m | 2.81, dd (11.0, 2.4) | 2.82, dd (13.3, 1.8) | 6′a | 4.17, d (14.2) | 4.17, d (12.3) | 4.18, m |
| 4b | 2.68, m | 2.68, t (11.0) | 2.68, dd (12.3, 5.9) | 6′b | 4.02, m | 4.04, m | 4.03, d (11.1) |
| 6 | 5.22, br. s | 5.28, br. s | 5.30, br. s | Rha-1″ | 6.41, br. s | 6.41, br. s | 6.41, br. s |
| 7a | 1.87, m | 1.89, m (2H) | 1.90, m | 2″ | 4.86, m | 4.85, m | 4.85, m |
| 7b | 1.43, m | | 1.52, m | 3″ | 4.62, m | 4.63, m | 4.63, m |
| 8 | 1.85, m | 1.79, m | 1.86, m | 4″ | 4.35, m | 4.37, m | 4.36, m |
| 9 | 1.30, m | 1.32, m | 1.38, m | 5″ | 4.93, m | 4.95, m | 4.94, m |
| 11a | 2.56, t (13.5) | 2.53, t (14.0) | 2.64, t (13.5) | 6″ | 1.58, d (5.2) | 1.59, d (5.9) | 1.59, d (5.6) |
| 11b | 2.30, dd (14.4, 5.6) | 2.31, dd (14.5, 5.7) | 2.25, dd (13.5, 6.3) | Rha-1‴ | 5.84, br. s | 5.84, br. s | 5.83, br. s |
| 14 | 1.41, m | 1.23, m | 1.58, m | 2‴ | 4.51, m | 4.51, m | 4.53, m |
| 15a | 2.09, m | 2.20, m | 2.25, m | 3‴ | 4.55, m | 4.56, m | 4.55, m |
| 15b | 1.64, m | 1.67, m | 2.07, m | 4‴ | 4.44, m | 4.45, m | 4.43, m |
| 16 | 4.88, m | 4.84, m | 6.54, s | 5‴ | 4.92, m | 4.93, m | 4.93, m |
| 17 | 2.94, t (7.5) | 3.45, d (10.2) | | 6‴ | 1.58, d (5.2) | 1.59, d (5.9) | 1.59, d (5.6) |
| 18 | 1.15, s | 0.95, s | 1.33, s | Rha-1⁗ | 6.29, br. s | 6.30, br. s | 6.29, br. s |
| 19 | 1.06, s | 1.07, s | 1.07, s | 2⁗ | 4.90, m | 4.90, m | 4.90, m |
| 20 | 2.20, dd (13.2, 6.5) | | | 3⁗ | 4.52, m | 4.53, m | 4.53, m |
| 21 | 1.53, d (6.7) | 1.74, s | 2.32, s | 4⁗ | 4.30, m | 4.31, m | 4.31, m |
| 23a | 2.05, m | 2.20, m (2H) | | 5⁗ | 4.36, m | 4.38, m | 4.36, m |
| 23b | 1.52, m | | | 6⁗ | 1.75, d (6.1) | 1.76, d (6.2) | 1.76, d (6.2) |
| 24a | 2.04, m | 1.89, m | | Glc-1′′′′′ | 4.80, d (7.7) | 4.84, d (7.8) | |
| 24b | 1.66, m | 1.45, m | | 2′′′′′ | 3.60, br. d (9.1) | 3.60, dd (9.4, 5.4) | |
| 25 | 1.92, m | 1.93, m | | 3′′′′′ | 4.24, m | 4.26, m | |
| 26a | 3.94, dd (9.1, 7.1) | 3.95, dd (9.4, 7.1) | | 4′′′′′ | 4.21, m | 4.25, m | |
| 26b | 3.60, dd (9.1, 5.8 ) | 3.59, dd (9.4, 5.4) | | 5′′′′′ | 3.94, m | 3.96, m | |
| 27 | 0.97, d (6.5) | 1.01, d (6.6) | | 6′′′′′a | 4.54, m | 4.58, m | |
| | | | | 6′′′′′b | 4.38, m | 4.40, m | |

[a]Assignments based on 2D NMR spectra; [b]Recorded at 400 MHz; [c]Recorded at 500 MHz.

determined as $C_{57}H_{92}O_{27}$. The $^{1}$H NMR spectrum of **1** (Table 1) showed signals of four steroid methyl groups at $\delta_H$ 0.97 (3H, d, $J$ = 6.5 Hz, Me-27), 1.06 (3H, s, Me-19), 1.15 (3H, s, Me-18), and 1.53 (3H, d, $J$ = 6.7 Hz, Me-21), an olefinic proton at $\delta_H$ 5.22 (1H, br. s), as well as signals for five anomeric proton signals at $\delta_H$ 4.80 (1H, d, $J$ = 7.7 Hz, H-1'''''), 4.93 (1H, d, $J$ = 7.2 Hz, H-1'), 5.84 (1H, br. s, H-1'''), 6.29 (1H, br. s, H-1''''), and 6.41 (1H, br. s, H-1''). The three methyl carbon signals at $\delta_C$ 18.7, 18.9, and 18.5 and their corresponding proton signals at $\delta_H$ 1.58 (3H, d, $J$ = 5.2 Hz, H-6''), 1.58 (3H, d, $J$ = 5.2 Hz, H-6'''), and 1.75 (3H, d, $J$ = 6.1 Hz, H-6'''') indicated that **1** had three deoxy sugars. The monosaccharides of the acidic hydrolysate of **1** were identified as D-glucose and L-rhamnose on the basis of GC analysis and comparison with authentic standards. The above $^{1}$H NMR and chemical data, together with an acetalic carbon signal at $\delta_C$ 110.9 in the $^{13}$C NMR spectrum[15] and a positive coloration with Ehrlich reagent,[16,17] indicated **1** to be a furostanol saponin with up to five monosaccharides. A comparison of the $^{1}$H and $^{13}$C spectroscopic signals of the aglycone moiety of **1** with Proto-Pb (**5**)[8] indicated that the signals were similar except for the presence of a carbonyl group ($\delta_C$ 213.1). In the HMBC spectrum (Figure 1), the long-range correlations from $\delta_H$ 1.15 (Me-18, s) to $\delta_C$ 213.1 (C-12, s), 55.4 (C-13, s), 56.0 (C-14, d), and 54.9 (C-17, d) indicated that the carbonyl group was attached at C-12 of the aglycone of **1**. The configuration of the methyl group at C-25 is $R$ on the basis of the proton signals of C-26 at $\delta_H$ 3.94 (1H, 26-H$_a$) and 3.60 (1H, 26-H$_b$), and the difference ($\Delta_{ab}$) of the proton signals at C-26 was 0.34.[18,19] From the above evidence, the aglycone of **1** was identified as (25*R*)-furost-5-en-3*β*,22*α*,26-triol-12-one.

Comparison of the carbon chemical shift thus assigned with

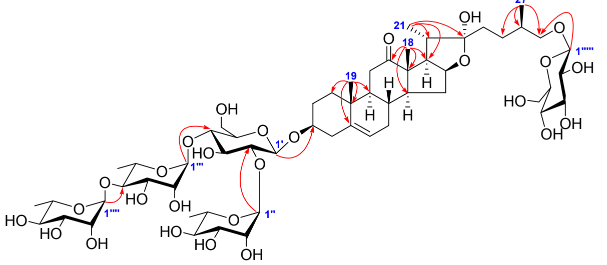

**Figure 1.** Selected HMBC correlations of conpound **1**.

those of the reference methyl glycosides,[15,19] taking into account the known effects of *O*-glycosylation, indicated that **1** contained a terminal *β*-D-glucopyranosyl units, two terminal *α*-L-rhamnopyranosyl unit, a C-4 substituted *α*-L-rhamnopyranosyl unit, and a C-2 and C-4 disubstituted *β*-D-glucopyranosyl unit. The *β*-configuration of the anomeric protons of the glucopyranosyl residue were assigned based on its $J_{1H\text{-}2H}$ value ( $J$ = 7.2~7.7 Hz), while the anomeric configuration of the three rhamnopyranosyls were determined as *α*-oriented on the ground the chemical shift values of the C-3″, C-5″, C-3‴, C-5‴, C-3⁗, and C-5⁗ with those of the corresponding carbons of methyl *α*- and *β*-rhamnopyranoside.[20,21] In the HMBC spectrum, a correlation peak between the signals at $\delta_H$ 4.80 (H-1 of terminal glucosyl) and $\delta_C$ 75.4 (C-26 of aglycon) implied that one glucose unit was attached at C-26 of the aglycon, which is a structural feature most frequently encountered in the plant furostanol saponins.[15] Consequently, a tetraglycoside was assumed to be located at C-3 of the aglycon. The sequence of the tetrasaccharide, which was the same as the known compounds **5**–**12**, was established from the further HMBC correlations: H-1′ ($\delta_H$ 4.93) of Glc with C-3 ($\delta_C$ 77.8) of the aglycone, H-1″ ($\delta_H$ 6.41) of 2′-Rha

**Table 2. $^{13}C$ NMR spectral data for compounds 1–3 ($C_5D_5N$)**

| Pos. | **1**[a] | **2**[b] | **3**[b] | Pos. | **1**[a] | **2**[b] | **3**[b] |
|---|---|---|---|---|---|---|---|
| 1 | 37.2, $CH_2$ | 37.1, $CH_2$ | 37.0, $CH_2$ | Glc-1′ | 100.4, CH | 100.4, CH | 100.4, CH |
| 2 | 30.0, $CH_2$ | 30.0, $CH_2$ | 29.9, $CH_2$ | 2′ | 78.0, CH | 77.9, CH | 78.0, CH |
| 3 | 77.8, CH | 77.9, CH | 77.9, CH | 3′ | 77.7, CH | 77.7, CH | 77.8, CH |
| 4 | 38.8, $CH_2$ | 38.8, $CH_2$ | 38.8, $CH_2$ | 4′ | 77.8, CH | 77.8, CH | 77.9, CH |
| 5 | 140.6, C | 140.7, C | 141.1, C | 5′ | 77.1, CH | 77.1, CH | 77.1, CH |
| 6 | 121.7, CH | 121.6, CH | 121.5, CH | 6′ | 61.2, $CH_2$ | 61.3, $CH_2$ | 61.3, $CH_2$ |
| 7 | 31.9, $CH_2$ | 31.9, $CH_2$ | 31.4, $CH_2$ | Rha-1″ | 102.3, CH | 102.2, CH | 102.2, CH |
| 8 | 31.0, CH | 30.8, CH | 30.2, CH | 2″ | 72.6, CH | 72.5, CH | 72.5, CH |
| 9 | 52.4, CH | 52.4, CH | 53.8, CH | 3″ | 72.7, CH | 72.6, CH | 72.7, CH |
| 10 | 37.7, C | 37.8, C | 38.0, C | 4″ | 74.2, CH | 74.1, CH | 74.2, CH |
| 11 | 37.6, $CH_2$ | 37.7, $CH_2$ | 37.8, $CH_2$ | 5″ | 69.5, CH | 69.6, CH | 69.6, CH |
| 12 | 213.1, C | 212.8, C | 209.2, C | 6″ | 18.7, $CH_3$ | 18.7, $CH_3$ | 18.7, $CH_3$ |
| 13 | 55.4, C | 57.2, C | 61.3, C | Rha-1‴ | 102.3, CH | 102.3, CH | 102.3, CH |
| 14 | 56.0, CH | 54.4, CH | 56.2, CH | 2‴ | 72.9, CH | 72.9, CH | 72.9, CH |
| 15 | 31.9, $CH_2$ | 34.0, $CH_2$ | 31.8, $CH_2$ | 3‴ | 73.4, CH | 73.3, CH | 73.3, CH |
| 16 | 79.8, CH | 83.1, CH | 142.8, CH | 4‴ | 80.5, CH | 80.4 , CH | 80.4, CH |
| 17 | 54.9, CH | 56.1, CH | 150.7, C | 5‴ | 68.4, CH | 68.4, CH | 68.4, CH |
| 18 | 16.1, $CH_3$ | 14.0, $CH_3$ | 16.4, $CH_3$ | 6‴ | 18.9, $CH_3$ | 18.9, $CH_3$ | 18.8, $CH_3$ |
| 19 | 19.0, $CH_3$ | 18.9, $CH_3$ | 18.9, $CH_3$ | Rha-1⁗ | 103.4, CH | 103.3, CH | 103.3, CH |
| 20 | 41.4, CH | 103.1, C | 196.0, C | 2⁗ | 72.7, CH | 72.6, CH | 72.5, CH |
| 21 | 15.3, $CH_3$ | 11.6, $CH_3$ | 27.7, $CH_3$ | 3⁗ | 72.9, CH | 72.9, CH | 72.9, CH |
| 22 | 110.9, C | 153.2, C | | 4⁗ | 74.0, CH | 74.0, CH | 74.1, CH |
| 23 | 37.1, $CH_2$ | 23.8, $CH_2$ | | 5⁗ | 70.5, CH | 70.4, CH | 70.5, CH |
| 24 | 28.4, $CH_2$ | 31.4, $CH_2$ | | 6⁗ | 18.5, $CH_3$ | 18.5, $CH_3$ | 18.5, $CH_3$ |
| 25 | 34.3, CH | 33.6, CH | | Glc-1′′′′′ | 105.0, CH | 105.0, CH | |
| 26 | 75.4, $CH_2$ | 75.2, $CH_2$ | | 2′′′′′ | 75.3, CH | 75.0, CH | |
| 27 | 17.5, $CH_3$ | 17.3, $CH_3$ | | 3′′′′′ | 78.7, CH | 78.6, CH | |
| | | | | 4′′′′′ | 71.7, CH | 71.7, CH | |
| | | | | 5′′′′′ | 78.6, CH | 78.5, CH | |
| | | | | 6′′′′′ | 62.9, $CH_2$ | 62.9, $CH_2$ | |

[a]Recorded at 100 MHz; [b]Recorded at 125 MHz.

with C-2′ ($\delta_C$ 78.0) of Glc, H-1‴ ($\delta_H$ 5.84) of 4′-Rha with C-4′ ($\delta_C$ 77.8) of Glc, and H-1⁗ ($\delta_H$ 6.29) of 4″-Rha with C-4‴ ($\delta_C$ 80.5) of 4′-Rha. Therefore, **1** was determined to be 26-*O*-*β*-D-glucopyranosyl-(25*R*)-3*β*,22*α*,26-trihydroxyfurost-5-en-12-one 3-*O*-*α*-L-rhamnopyranosyl-(1→4)-*α*-L-rhamnopyranosyl-(1→4)-[*α*-L-rhamnopyranosyl-(1→2)]-*β*-D-glucopyranoside, and named ypsilandroside P.

Compound **2** displayed a $[M - H]^-$ ion at *m/z* 1189.5645 (calcd. for $C_{57}H_{89}O_{26}$, 1189.5642) in the HRESIMS and gave a red color with Ehrlich's reagent. The NMR spectral data suggested **2** is a furostanol saponin closely related to **1**. It differed from **1** in the presence of one more olefinic functionality [$\delta_C$ 153.2 (s) and 103.1 (s)] in addition to the 5(6)-en group. Furthermore, the Me-21 methyl doublet signal observed at $\delta_H$ 1.53 ($J$ = 6.7 Hz) in the $^1H$ NMR spectrum of **1** was absent from **2**, but was replaced by a methyl singlet at $\delta_H$ 1.74. These data were suggestive of **2** being the corresponding $\Delta^{20(22)}$-furostanol saponin of **1,** which was confirmed by the mass difference of *m/z* = 18 and HMBC correlations. In the HMBC spectrum of **2**, the correlations of Me-21 ($\delta_H$ 1.74) with C-17 ($\delta_C$ 56.1), C-20 ($\delta_C$ 103.1), and C-22 ($\delta_C$ 153.2) were observed. Thus, the structure of **2** was established as 26-*O*-*β*-D-*β*-D-glucopyranosyl-(25*R*)-3*β*,26-dihydroxyfurost-5,20 (22)-diene-12-one 3-*O*-*α*-L-rhamnopyranosyl-(1→4)-*α*-L-rhamnopyranosyl-(1→4)-[*α*-L-rhamnopyranosyl-(1→2)]-*β*-D-glucopyranoside, and named ypsilandroside Q.

Compound **3** had a molecular formula of $C_{45}H_{68}O_{20}$, established by analysis of HRESIMS (*m/z* 963.3953 $[M + Cl]^-$, calcd. 963.3992) and $^{13}C$ NMR spectrum (45 signals). The $^1H$ NMR spectrum of **3** displayed two three-proton singlet signals at $\delta_H$ 1.07 (s) and 1.33 (s), indicating the presence of two angular methyl groups, and a methyl singlet at $\delta_H$ 2.32 (s) attached to a deshielding moiety, as well as four anomeric proton signals at $\delta_H$ 4.93 (1H, d, $J$ = 7.4 Hz), 5.83 (1H, br. s), 6.29 (1H, br. s), and 6.41 (1H, br. s). The existence of an *α*,*β*-unsaturated carbonyl group was verified by the IR (1657 $cm^{-1}$), UV [227 nm (log*ε* 2.8)], and $^{13}C$ NMR [$\delta_C$ 196.0 (C=O), 150.7 (C), and 142.8 (CH)] spectra. These spectral data and comparison with those of the known compound **8**[11] indicated that **3** differed from **8** by the presence of a carbonyl group ($\delta_C$ 209.2) instead of a methylene moiety at C-12 in the latter. The HMBC correlations of $\delta_H$ 1.33 (Me-18) with $\delta_C$ 209.2 (C-12, s), 61.3 (C-13, s), 56.2 (C-14, d), 150.7 (C-17, s) indicated that the location of the carbonyl group at C-12. Thus, the aglycone of **3** was identified as 3*α*-hydroxypregna-5,16-dien-12,20-dione. The $^1H$ and $^{13}C$ NMR shifts of the tetraglycoside moiety linked to C-3 of the pregnane were superimposable on those of **1**, **2**, and **5**–**12**. On the basis of all the information above, the structure of **3** was characterized as pregnane 5,16-dien-3*β*-ol-12,20-dione 3-*O*-*α*-L-rhamnopyranosyl-(1→4)-*α*-L-rhamnopyranosyl-(1→4)-[*α*-L-rhamnopyranosyl-(1→2)]-*β*-D-glucopyranoside, and named ypsilandroside R.

The cytotoxic activities of saponins **1**, **2**, and **12** against the growth of human tumor cell lines (A549 and HL-60) were evaluated. The results indicated that only compound **1** showed 86.4% inhibition to A549 cell lines and 75.9% inhibition to HL-60 cell lines at the tested concentration (10 *μ*M).

## Experimental Section

**General Experimental Procedures**. Optical rotations were measured on a Jasco P-1020 automatic digital polarimeter. UV spectra were measured using a Shimadzu UV-2401PC spectrophotometer. IR spectra were measured on a Bio-Rad FTS-135 spectrometer with KBr pellets. NMR spectra were run on Bruker AM-400 and DRX-500 instruments with TMS as internal standard. FAB-MS spectra were recorded on a VG Auto Spec-300 spectrometer, HRESIMS spectra were recorded on an API Qstar Pulsar instrument. Column chromatography (CC) was performed over silica gel (200–300 mesh, 10–40 $\mu$m, Qingdao Marine Chemical Co., China), Rp-18 (40–63 $\mu$m, Merck), and Sephadex LH-20 (GE Healthcare, Sweden). TLC was performed on HSGF254 (0.2 mm, Qingdao Marine Chemical Co., China) or Rp-18 $F_{254}$ (0.25 mm, Merck). Semi-preparative HPLC was run on Agilent 1100 liquid chromatograph with diode array detector (DAD) setting at 200nm and 254 nm, ZORBAX SB-C18 (5 $\mu$m) column (25 cm × 9.4 mm i.d.). GC analysis was performed on a Shimadzu GC-2010 gas chromatograph equipped with an $H_2$ flame ionization detector.

**Plant Material.** The plant material of *Y. thibetica* was collected in November 2006 from Luding County, Sichuan Province, China, and identified by Prof. Xin-Qi Chen, Institute of Botany, Chinese Academy of Sciences, Beijing. A voucher specimen (No. HY0002) was deposited at the State Key Laboratory of Phytochemistry and Plant Resources in West China.

**Extraction and Isolation.** The air-dried whole plants of *Y. thibetica* (10 kg) were extracted three times with 70% EtOH (50 L × 3) under reflux for a total of 6 h and the combined extract was concentrated under reduced pressure. Then the concentrated extract was loaded onto a macroporous resin column (YWD-3F) and eluted successively with $H_2O$, 40% EtOH (F1 fraction), 70% EtOH (F2 fraction), and 95% EtOH (F3 fraction), respectively. The 40% EtOH elutes were evaporated to dryness. Fraction F1 (33 g) was fractioned by silica gel column and eluted with a gradient of $CHCl_3$-MeOH-$H_2O$ (8:2:0.2→7:3:0.5, v/v) to get two subfractions (F11 and F12). Fraction F11 was subjected to column chromatography on Rp-18 gel (MPLC, MeOH-$H_2O$ 4:6→6.5:3.5) and semi-preparative HPLC (MeOH-$H_2O$ 38:62 v/v; flow rate: 3 $mL.min^{-1}$) to obtain **3** (14 mg), **4** (28 mg), and **8** (19 mg). Fraction F12 was chromatographed over Rp-18 gel (MPLC, MeOH-$H_2O$ 3:7→7:3) and semi-preparative HPLC (MeCN-$H_2O$ 20:80→35:65 v/v; flow rate: 3 $mL.min^{-1}$) to yield **1** (17 mg), **2** (8 mg), **5** (43 mg), **6** (80 mg), **7** (23 mg), **9** (20 mg), **10** (17 mg), **11** (18 mg), and **12** (24 mg).

**Ypsilandroside P (1):** white amorphous powder; $[\alpha]_D^{24}$ −65.0 (*c* 0.26, MeOH); IR (KBr) $\nu_{max}$ 3431, 2934, 1706, 1640, 1453, 1381, 1130, 1044, 985, 911, 839, 804 $cm^{-1}$ (intensity: 839 > 911); $^1H$ and $^{13}C$ NMR data see Tables 1 and 2; negative FABMS *m/z* 1208 $[M]^-$, 1062 $[M - 146]^-$, 915 $[M - H - 2 \times 146]^-$, 769 $[M - H - 3 \times 146]^-$; negative ion HRESIMS *m/z* 1207.5736 (calcd. for $C_{57}H_{91}O_{27}$ $[M - H]^-$, 1207.5747).

**Ypsilandroside Q (2):** white amorphous powder; $[\alpha]_D^{24}$ −66.8 (*c* 0.47, MeOH); IR (KBr) $\nu_{max}$ 3426, 2933, 1707, 1640, 1453, 1382, 1131, 1043, 984, 911, 841, 804 $cm^{-1}$ (intensity: 841 > 911); $^1H$ and $^{13}C$ NMR data see Tables 1 and 2; negative FABMS *m/z* 1190 $[M]^-$, 1043 $[M - H - 146]^-$, 897 $[M - H - 2 \times 146]^-$; negative ion HRESIMS *m/z* 1189.5645 (calcd. for $C_{57}H_{89}O_{26}$ $[M - H]^-$, 1189.5642).

**Ypsilandroside R (3):** white amorphous powder; $[\alpha]_D^{22}$ −58.3 (*c* 0.12, MeOH); UV (MeOH) $\lambda_{max}$ (log $\varepsilon$) 227 (2.8) nm; IR (KBr) $\nu_{max}$ 3418, 2934, 1713, 1657, 1376, 1132, 1053, 983 $cm^{-1}$; $^1H$ and $^{13}C$ NMR data see Tables 1 and 2; negative FABMS *m/z* 927 $[M - H]^-$, 781 $[M - H - 146]^-$, 635 $[M - H - 2 \times 146]^-$; negative ion HRESIMS *m/z* 963.3953 (calcd. for $C_{45}H_{68}O_{20}Cl$ $[M + Cl]^-$, 963.3992).

**Acid Hydrolysis of Compounds 1–3 and GC Analysis.** Compounds **1**–**3** (4 mg each) were refluxed with 4 M TFA-dioxane (1:1 v/v, 2 mL) on water bath for 4h. The reaction mixture was neutralized with 1 M NaOH and filtered. The filtrate was extracted with $CHCl_3$ and $H_2O$. The $H_2O$-soluble fraction was evaporated to dryness. The dried sugar residues was diluted in 1 mL pyridine without water and treated with 0.5 mL trimethyl-chlorsilan (TMCS) and stirred at 60°C for 5 min. After drying the solution with a stream of $N_2$, the residue was extracted with ether (1 mL). The ether layer was analyzed by GC under the following conditions: column, SGE AC-10 quartz capillary column (30 m × 0.32 mm × 0.25 $\mu$m); column temperature 180–280°C; programmed increase, 3 °C/min; carrier gas, $N_2$ (2 ml/min); injector and detector temperature, 250°C; injection volume, 2 $\mu$L; split ratio, 1/50. Peaks of the hydrolysate were detected by comparison with retention times of authentic samples of glucose and rhamnose after treatment with trimethyl-chlorsilan (TMCS) in pyridine. The absolute configurations of the sugar residues were determined to be L-rhamnose ($t_R$ 7.67 min) and D-glucose ($t_R$ 14.22 min).

**Cell-Growth Inhibition Assay.** Growth inhibition of compounds on tumor cells was determined by microculture 3-(4,5-dimethylthiazol-2-yl)-2,5-diphenyl-2H-tetrazolium hydrobromide (MTT) assay.[22]

## Electronic Supplementary Material

Supplementary material is available in the online version of this article at http://dx.doi.org/ 10.1007/s13659-011-0039-z and is accessible for authorized users.

## Acknowledgments

This work was financially supported by National Natural Science Funding of China (No. 31170333), the Natural Science Foundation of Yunnan Province (No. 2009CC019), the West Light Foundation of the Chinese Academy of Sciences (No. 2908025712W1), and a fund (No.540806321211) of State Key Laboratory of Phytochemistry and Plant Resources in West China, Germplasm Bank of Wild Species and CAS Innovation Program of Kunming Institute of Botany.

**Open Access** This article is distributed under the terms of the Creative Commons Attribution License which permits any use, distribution, and reproduction in any medium, provided the original author(s) and source are credited.

## Electronic supplementary material

Supplementary material, approximately 1.44 MB.
