# Supplementary material for: Steroidal and pregnane glycosides from Ypsilandra thibetica
Source: Nat Prod Bioprospect. 2012 Jan 14;2(1):11–5. doi: 10.1007/s13659-011-0039-z (PMC4131569; doi:10.1007/s13659-011-0039-z)

Electronic Supplementary Material

Steroidal and pregnane glycosides from *Ypsilandra thibetica*

Hai-Yang LIU,* Chang-Xiang CHEN, Yi LU, Jun-Yun YANG, and Wei NI

State Key Laboratory of Phytochemistry and Plant Resources in West China, Kunming Institute of Botany, Chinese Academy of Sciences, Kunming 650201, China

Received 27 November 2011; Accepted 26 December 2011

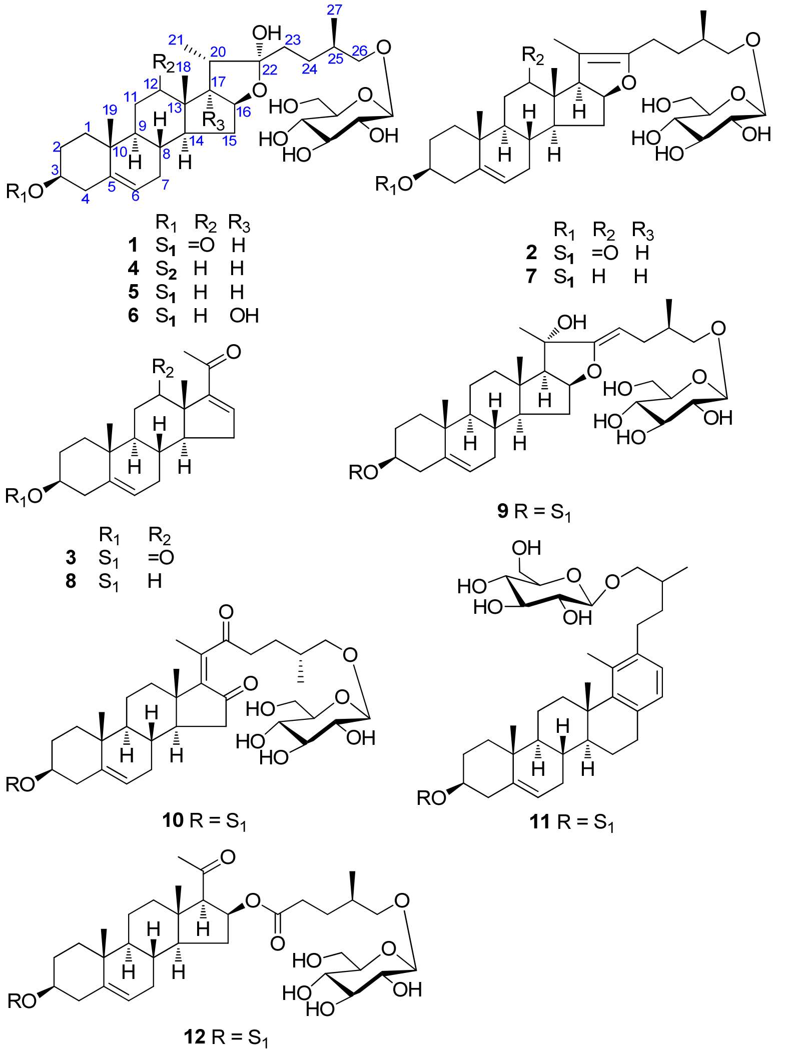

S$_1$ = α-L-Rha (1→2)-[α-L-Rha (1→4)-α-L-Rha(1→4)]-β-D-Glc-
S$_2$ = α-L-Rha (1→4)-β–D-Glc-

Structures of compounds **1–12**

*To whom correspondence should be addressed. E-mail: haiyangliu@mail.kib.ac.cn

Table of contents

Figure S1. ^{1}H NMR spectrum of ypsilandroside P (**1**) in C_5D_5N.

Figure S2. ^{13}C NMR spectrum of ypsilandroside P (**1**) in C_5D_5N.

Figure S3. HSQC spectrum of ypsilandroside P (**1**) in C_5D_5N.

Figure S4. HMBC spectrum of ypsilandroside P (**1**) in C_5D_5N.

Figure S5. FAB-MS spectrum of ypsilandroside P (**1**) in C_5D_5N.

Figure S6. HR-ESI-MS spectrum of ypsilandroside P (**1**) in C_5D_5N.

Figure S7. ^{1}H NMR spectrum of ypsilandroside Q (**2**) in C_5D_5N.

Figure S8. ^{13}C NMR spectrum of ypsilandroside Q (**2**) in C_5D_5N.

Figure S9. HSQC spectrum of ypsilandroside Q (**2**) in C_5D_5N.

Figure S10. HMBC spectrum of ypsilandroside Q (**2**) in C_5D_5N.

Figure S11. FAB-MS spectrum of ypsilandroside Q (**2**) in C_5D_5N.

Figure S12. HR-ESI-MS spectrum of ypsilandroside Q (**2**) in C_5D_5N.

Figure S13. ^{1}H NMR spectrum of ypsilandroside R (**3**) in C_5D_5N.

Figure S14. ^{13}C NMR spectrum of ypsilandroside R (**3**) in C_5D_5N.

Figure S15. HSQC spectrum of ypsilandroside R (**3**) in C_5D_5N.

Figure S16. HMBC spectrum of ypsilandroside R (**3**) in C_5D_5N.

Figure S17. FAB-MS spectrum of ypsilandroside R (**3**) in C_5D_5N.

Figure S18. HR-ESI-MS spectrum of ypsilandroside R (**3**) in C_5D_5N.

Figure S1. ^{1}H NMR spectrum of ypsilandroside P (1) in C_5D_5N.

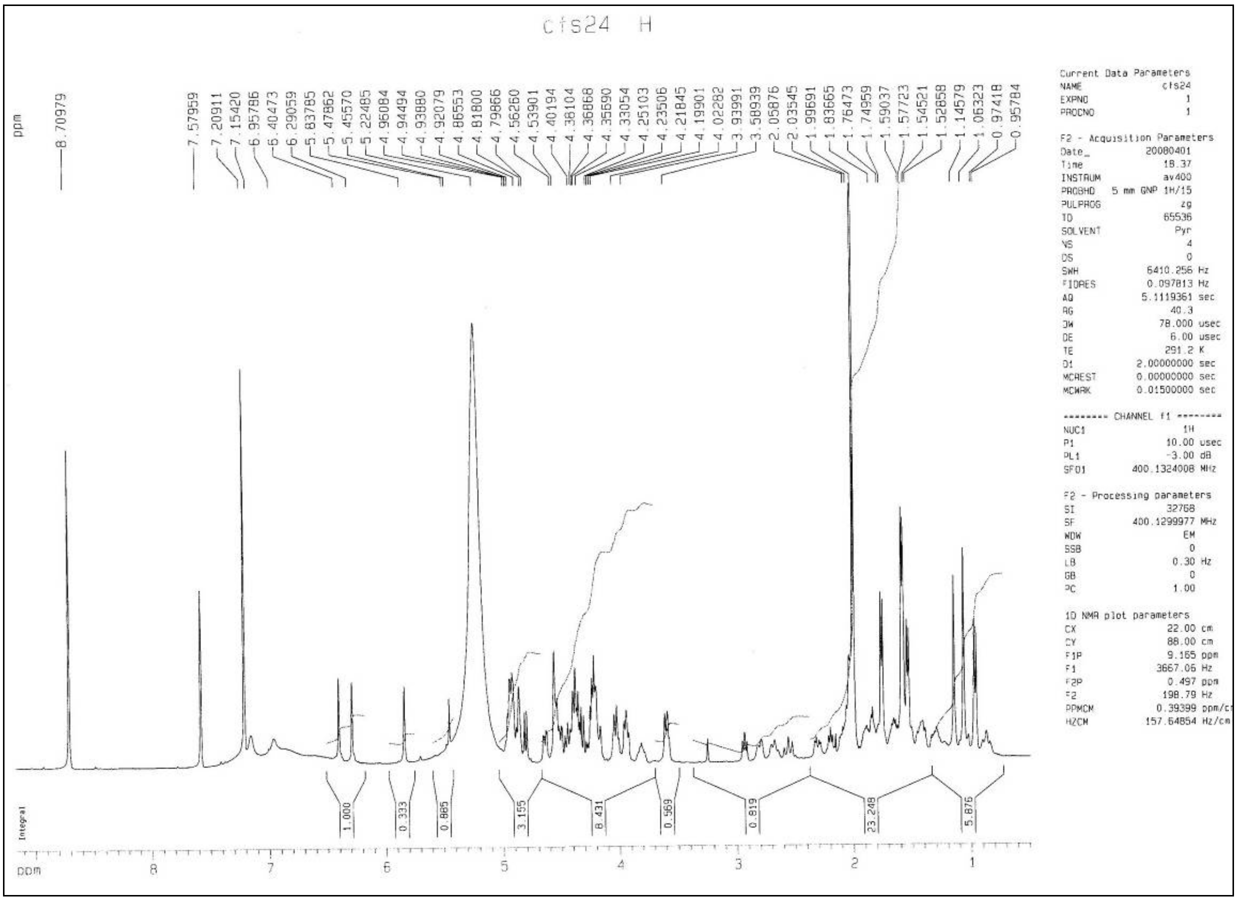

Figure S2. ^{13}C NMR spectrum of ypsilandroside P (1) in C_5D_5N.

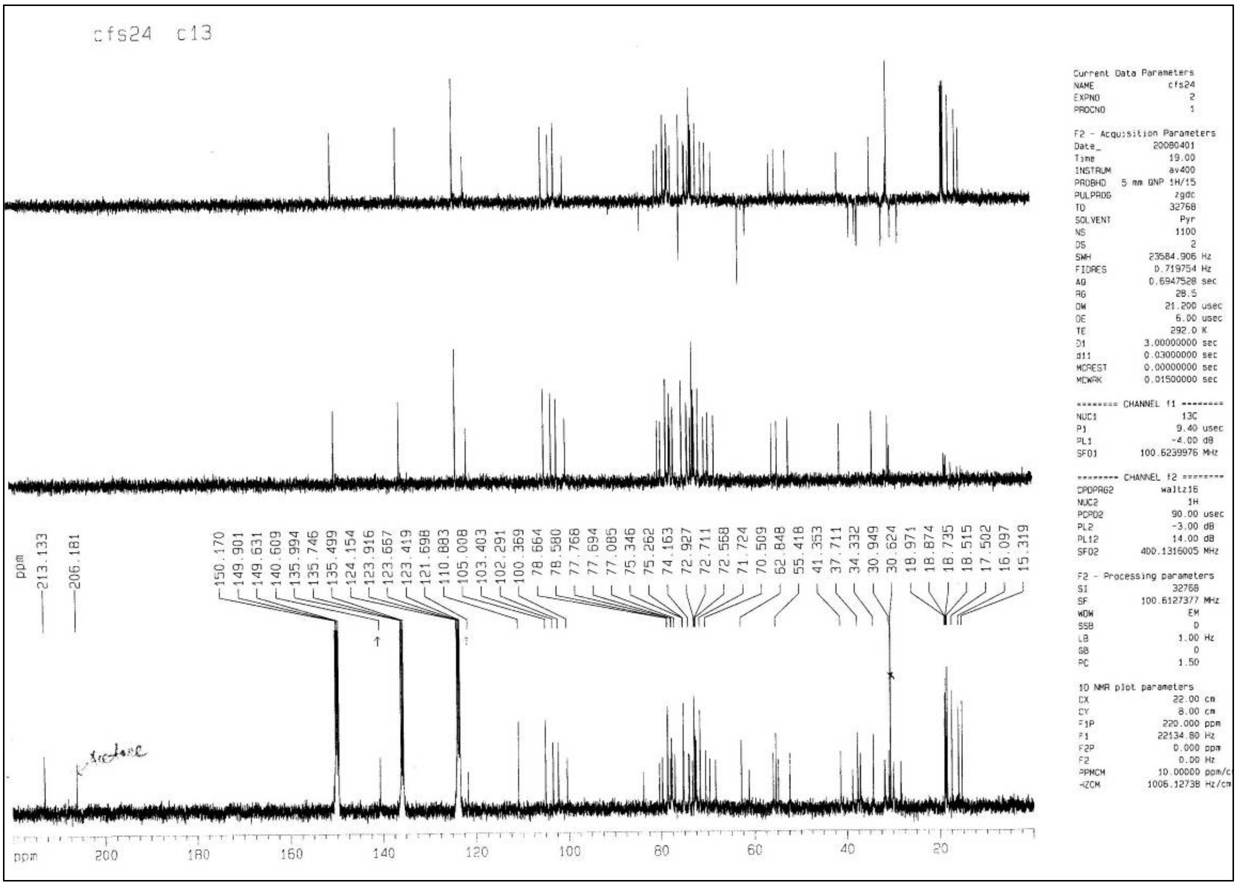

Figure S3. HSQC spectrum of ypsilandroside P (1) in C_5D_5N.

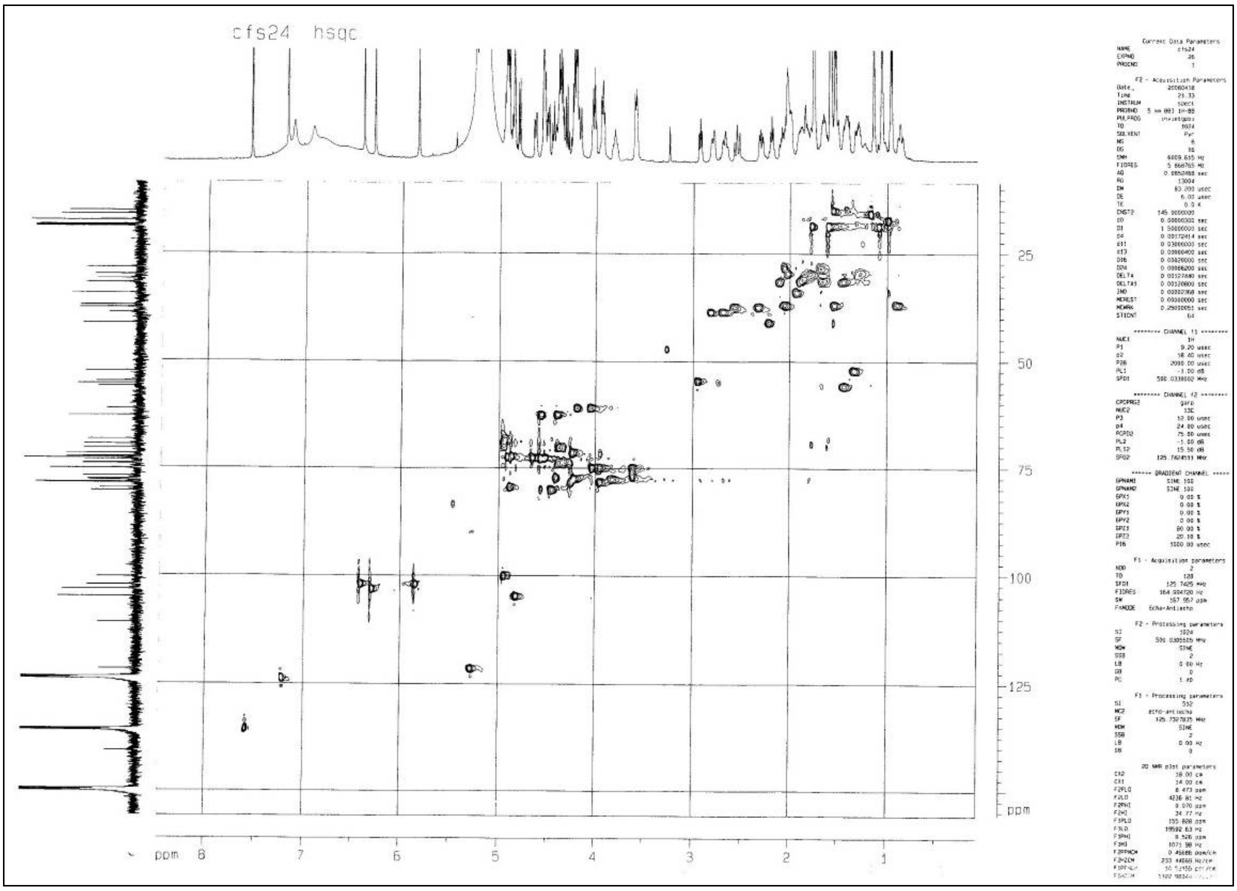

Figure S4. HMBC spectrum of ypsilandroside P (1) in C_5D_5N.

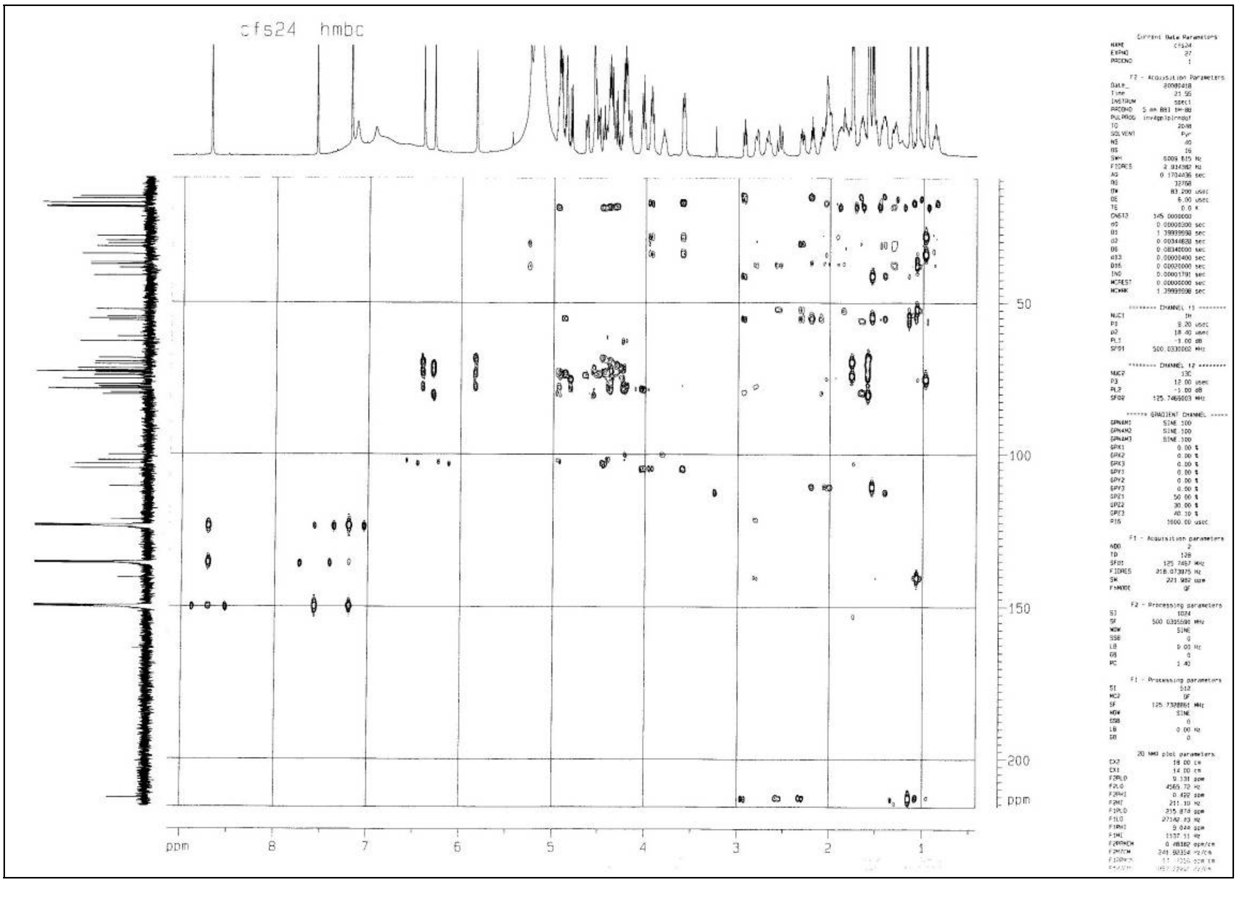

Figure S5. FAB-MS spectrum of ypsilandroside P (1) in C_5D_5N.

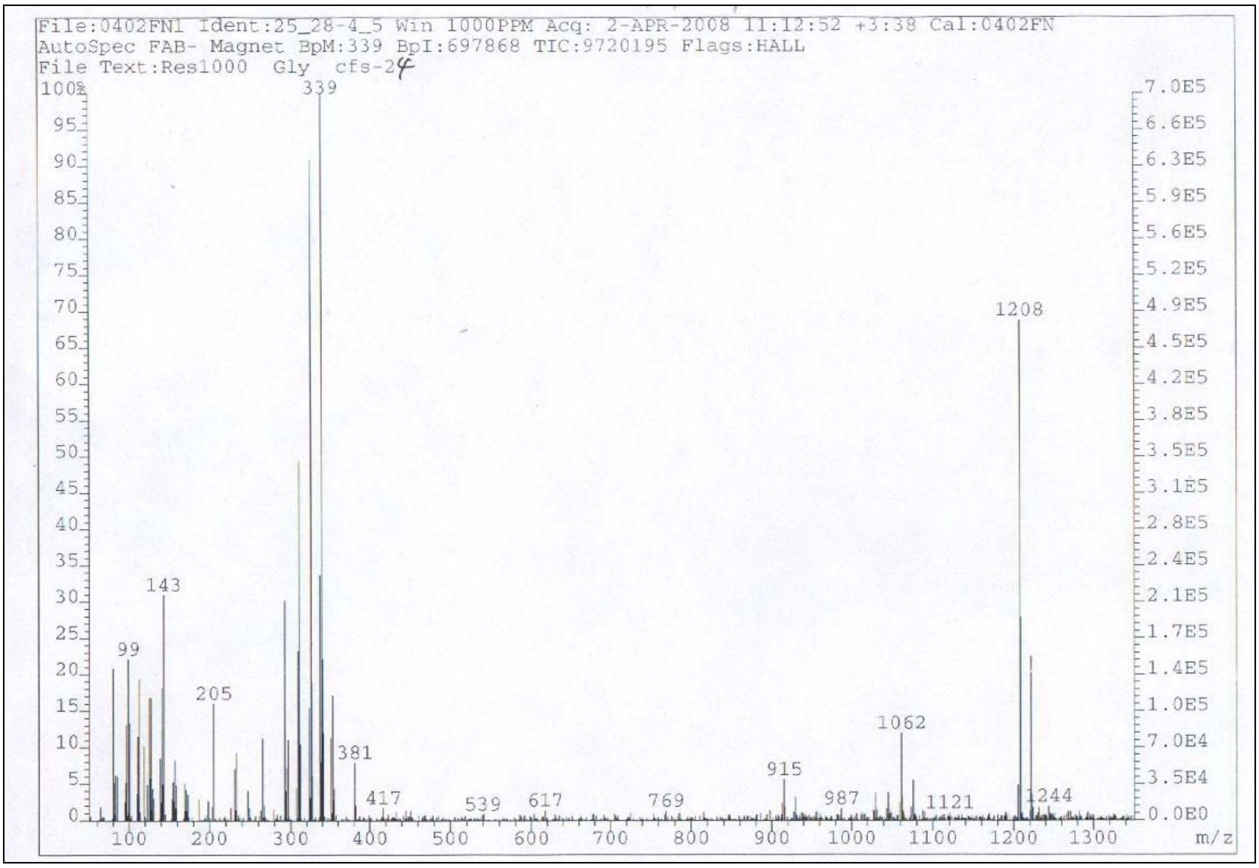

Figure S6. HR-ESI-MS spectrum of ypsilandroside P (1) in C_5D_5N.

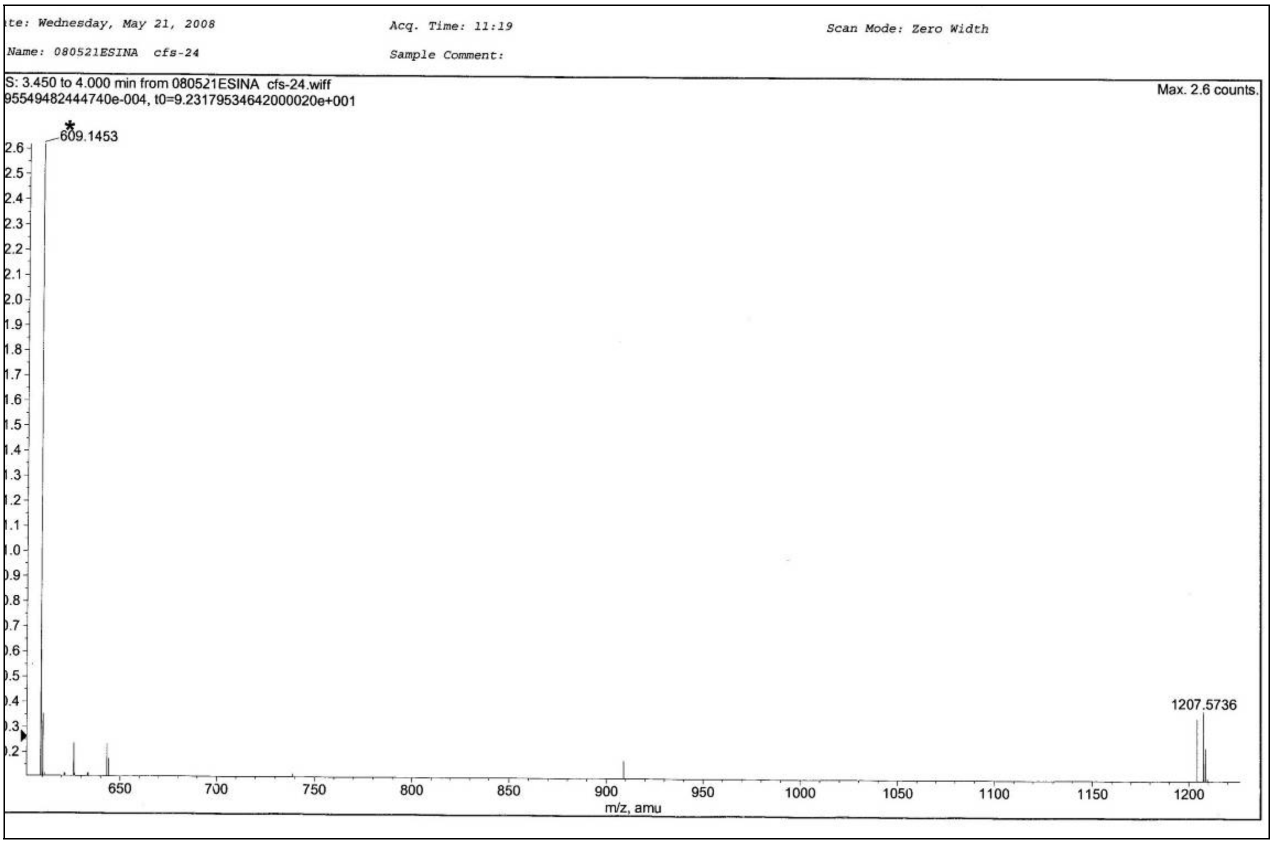

Figure S7. ^{1}H NMR spectrum of ypsilandroside Q (2) in C_5D_5N.

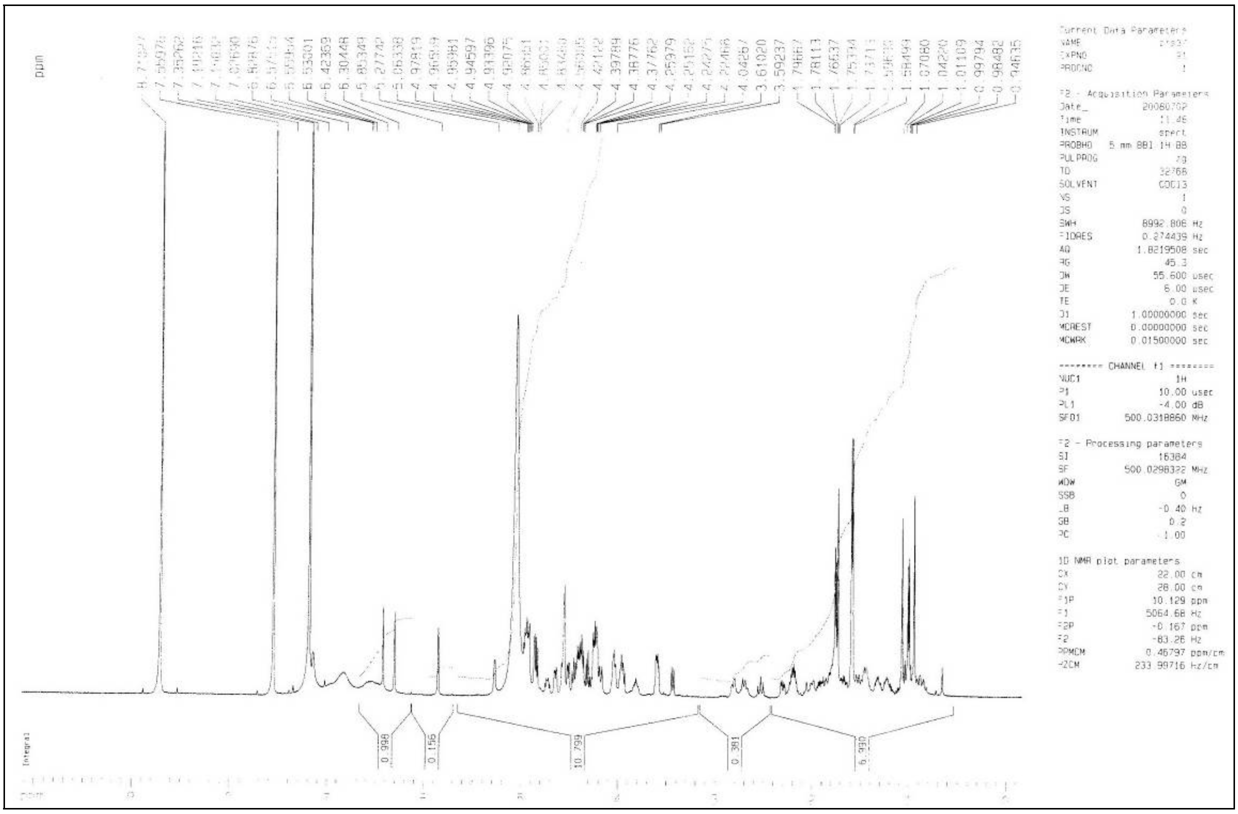

Figure S8. ^{13}C NMR spectrum of ypsilandroside Q (2) in C_5D_5N.

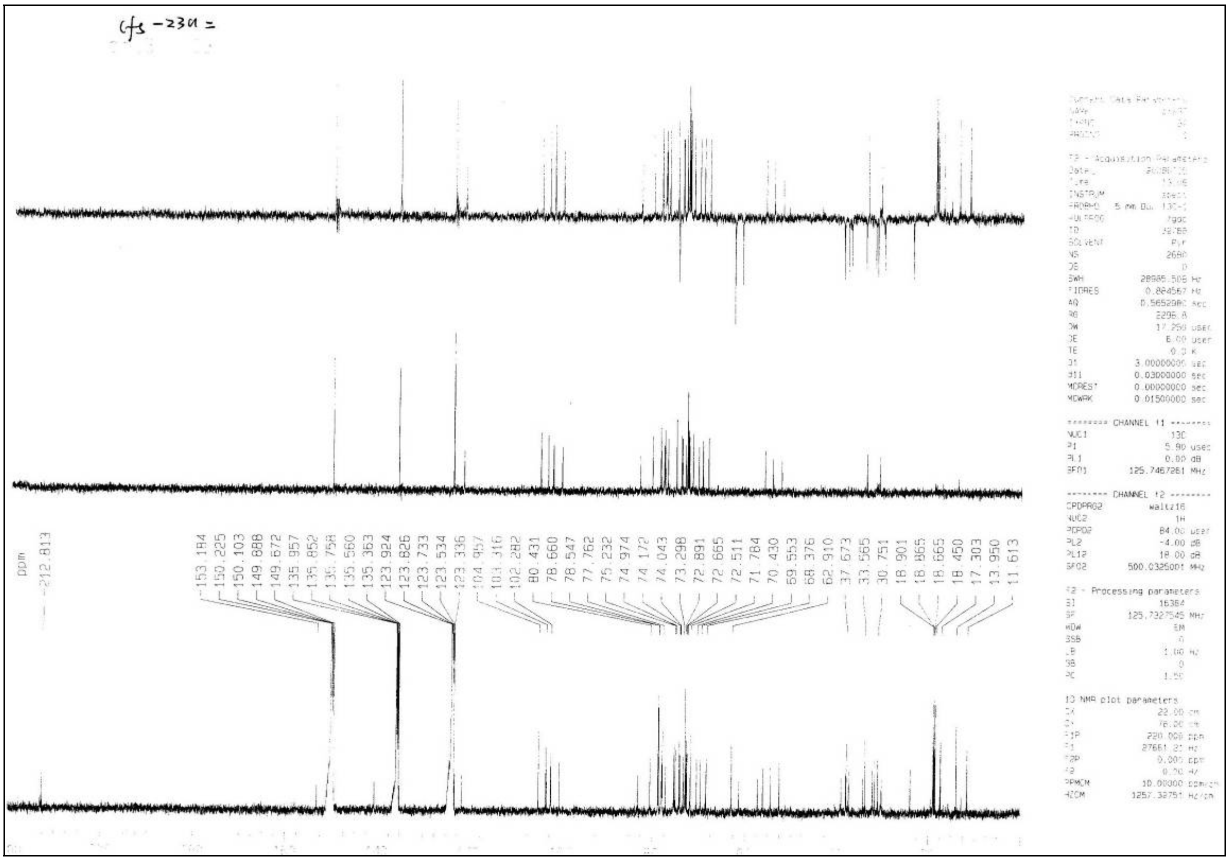

Figure S9. HSQC spectrum of ypsilandroside Q (2) in C_5D_5N.

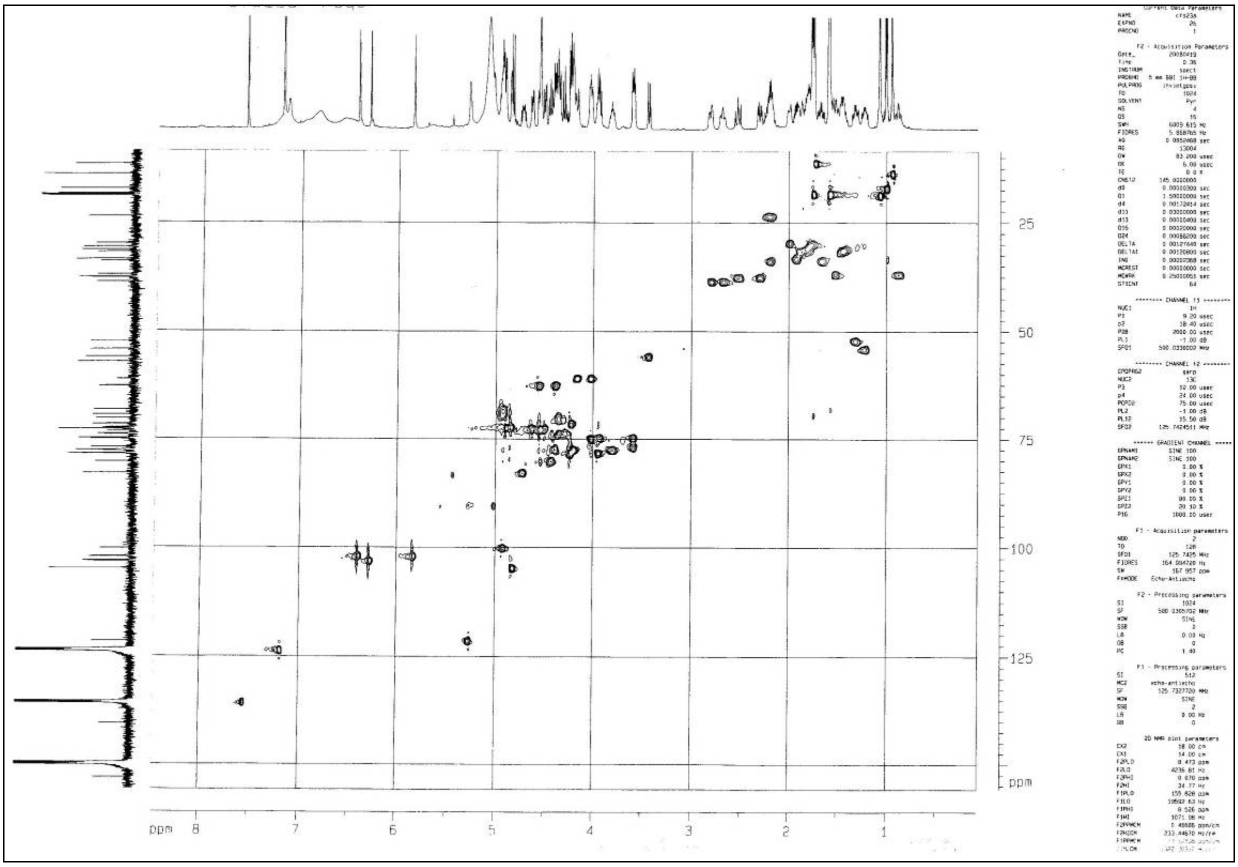

Figure S10. HMBC spectrum of ypsilandroside Q (2) in C_5D_5N.

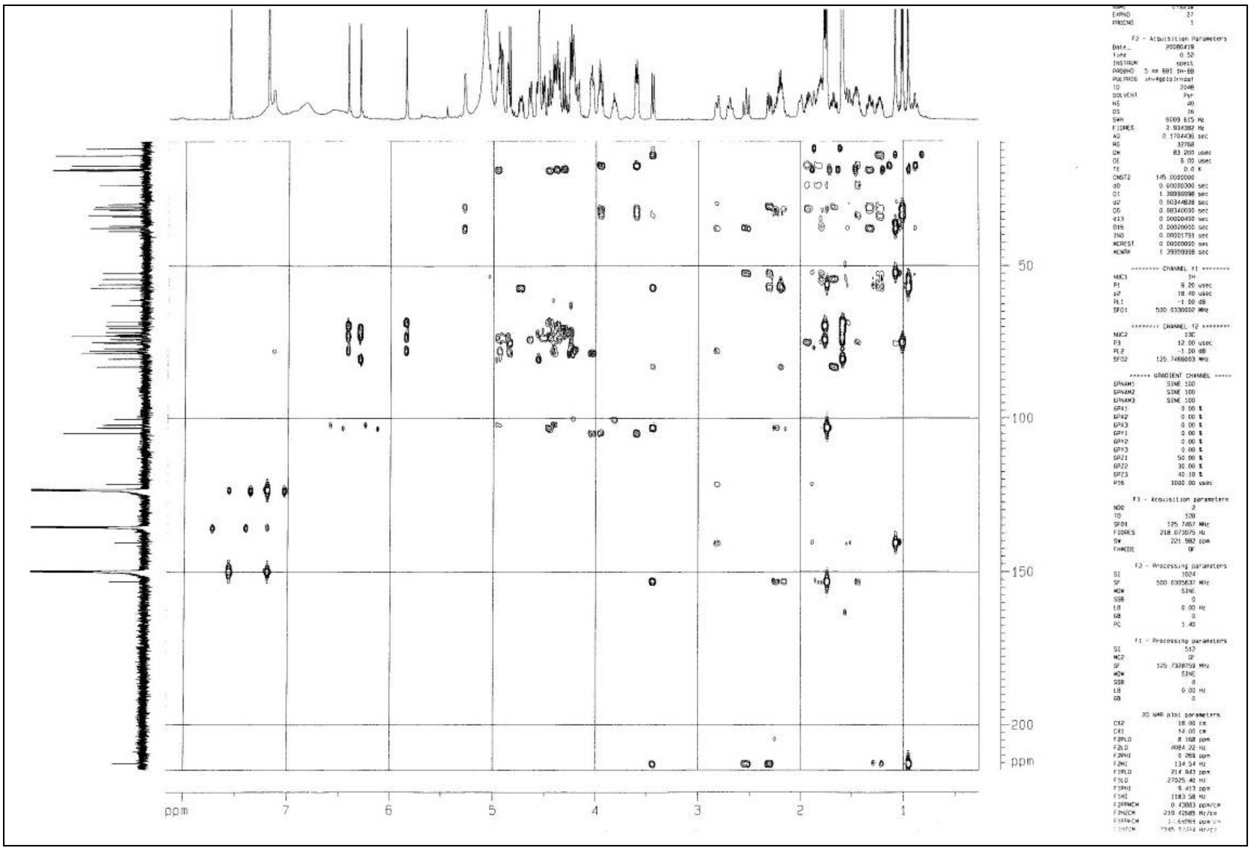

Figure S11. FAB-MS spectrum of ypsilandroside Q (2) in C_5D_5N.

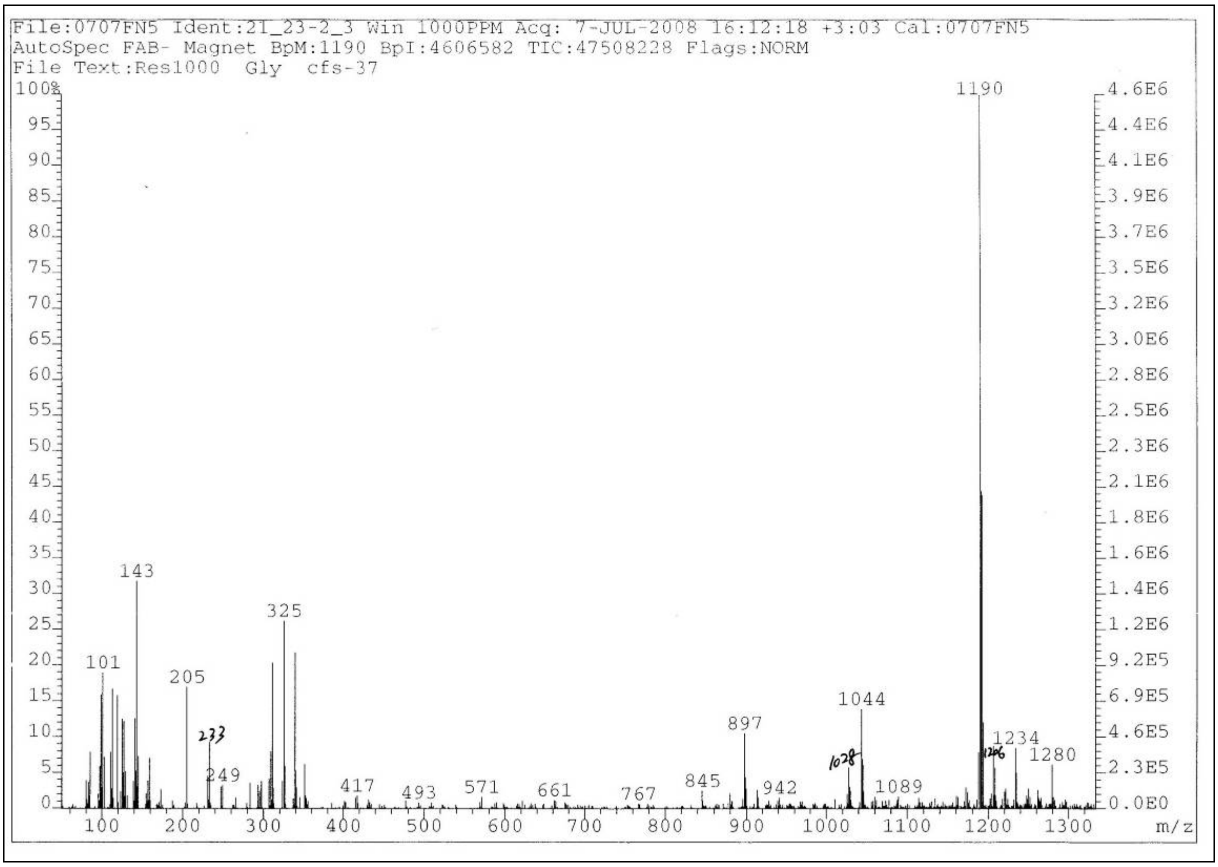

Figure S12. HR-ESI-MS spectrum of ypsilandroside Q (2) in C_5D_5N.

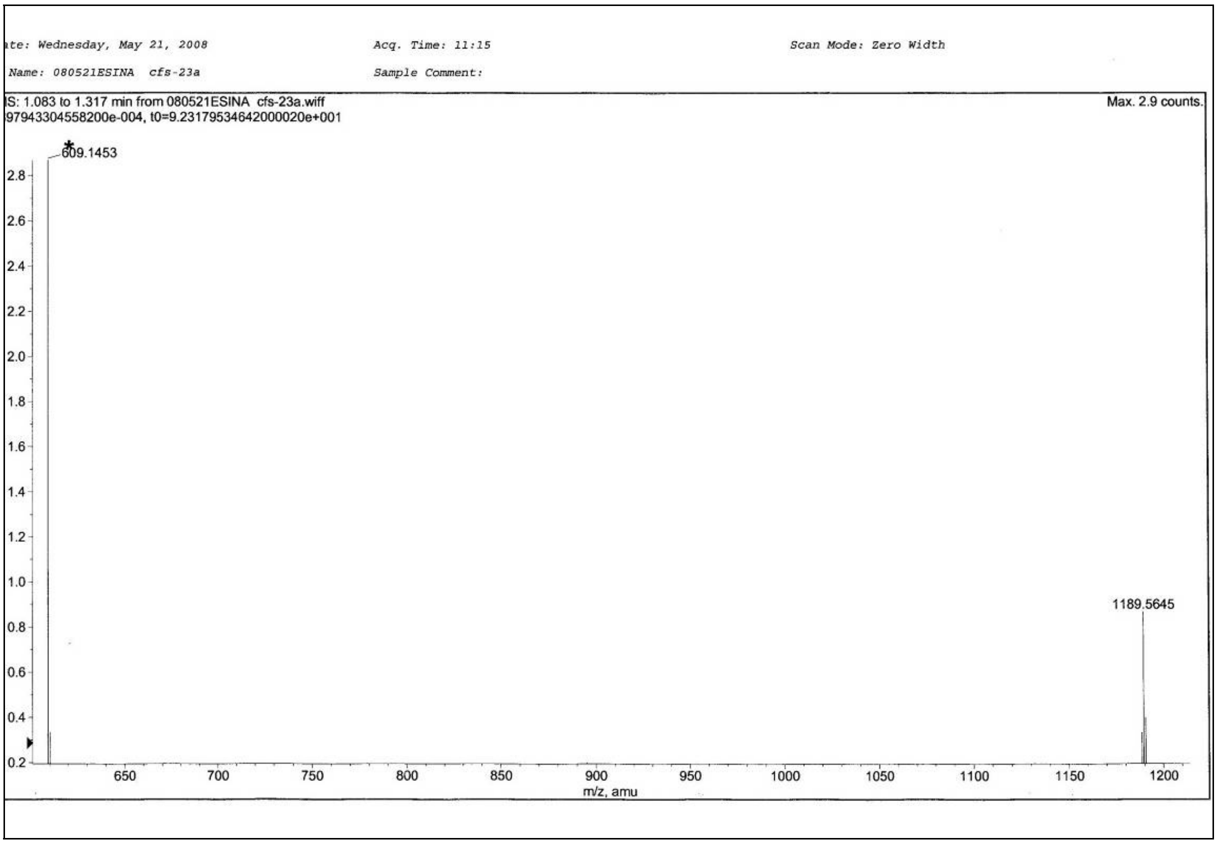

Figure S13. ^{1}H NMR spectrum of ypsilandroside R (3) in C_5D_5N.

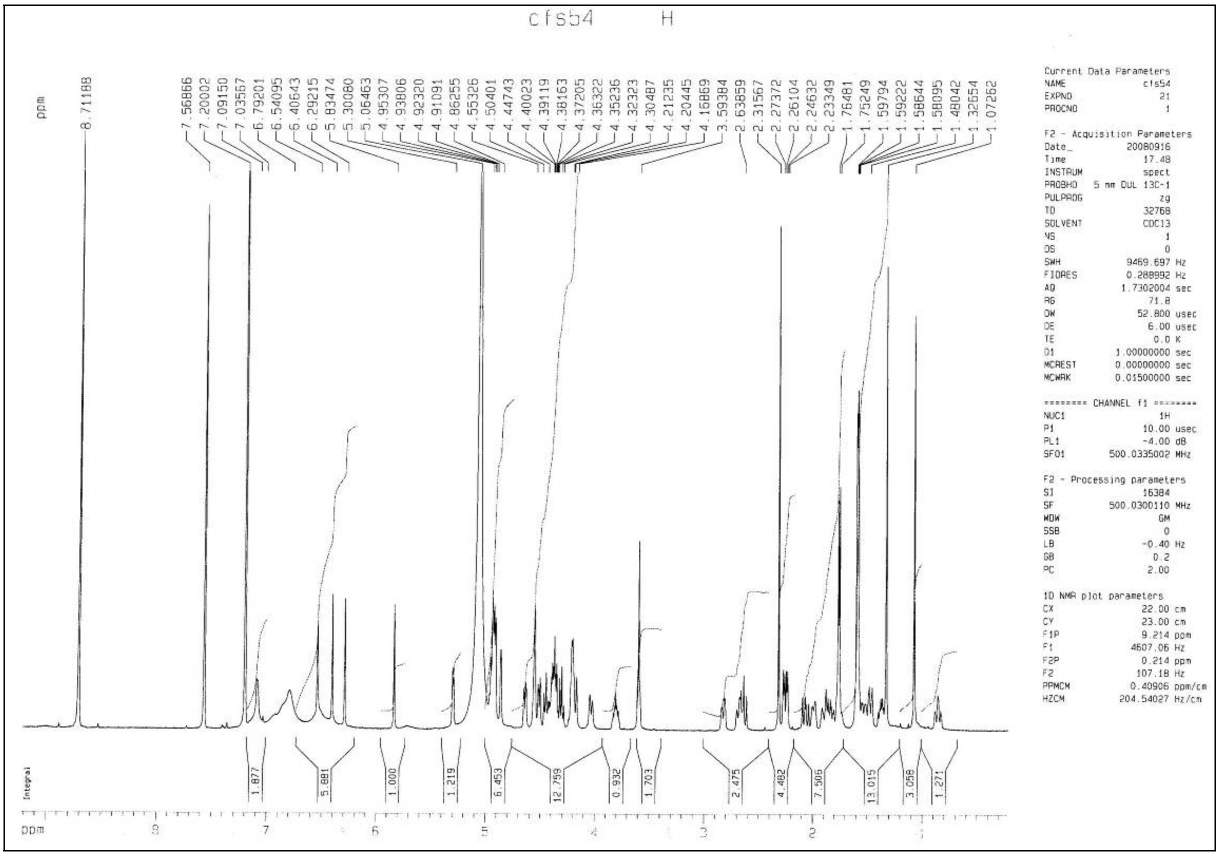

Figure S14. ^{13}C NMR spectrum of ypsilandroside R (3) in C_5D_5N.

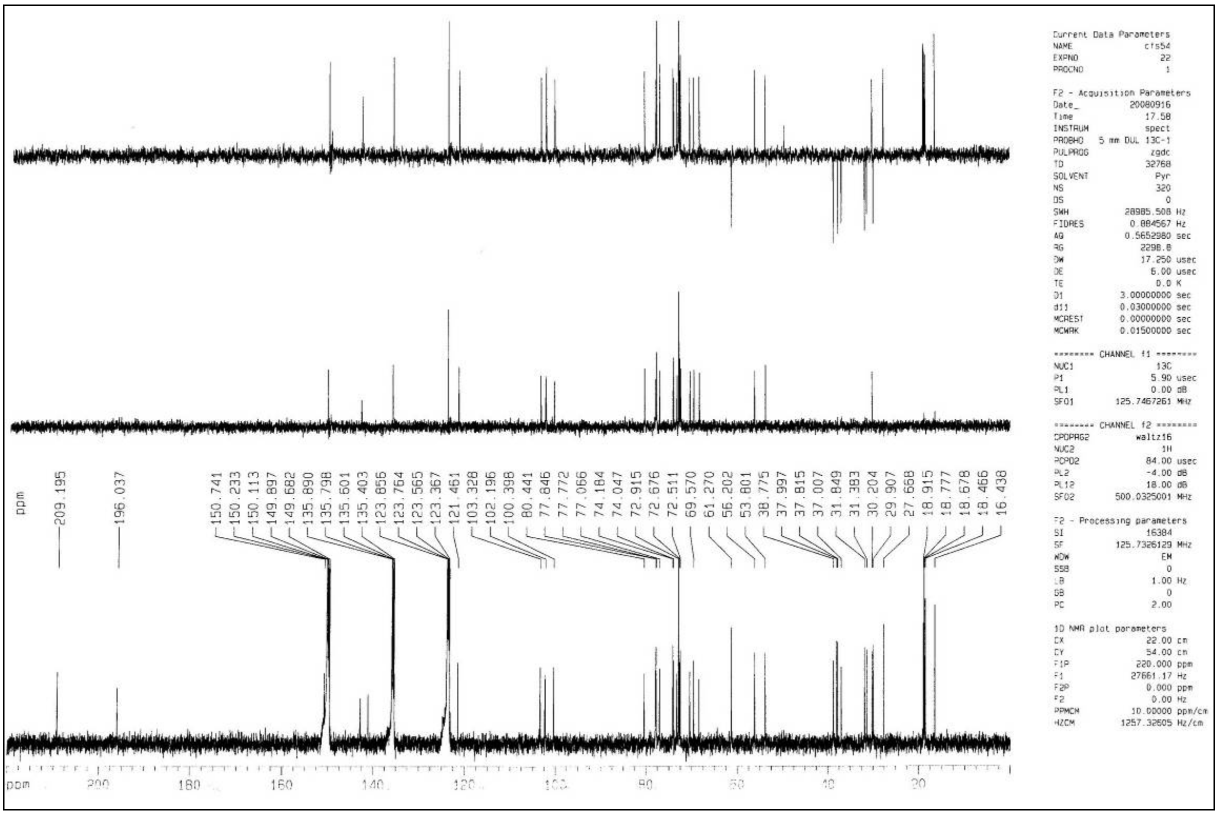

Figure S15. HSQC spectrum of ypsilandroside R (3) in C_5D_5N.

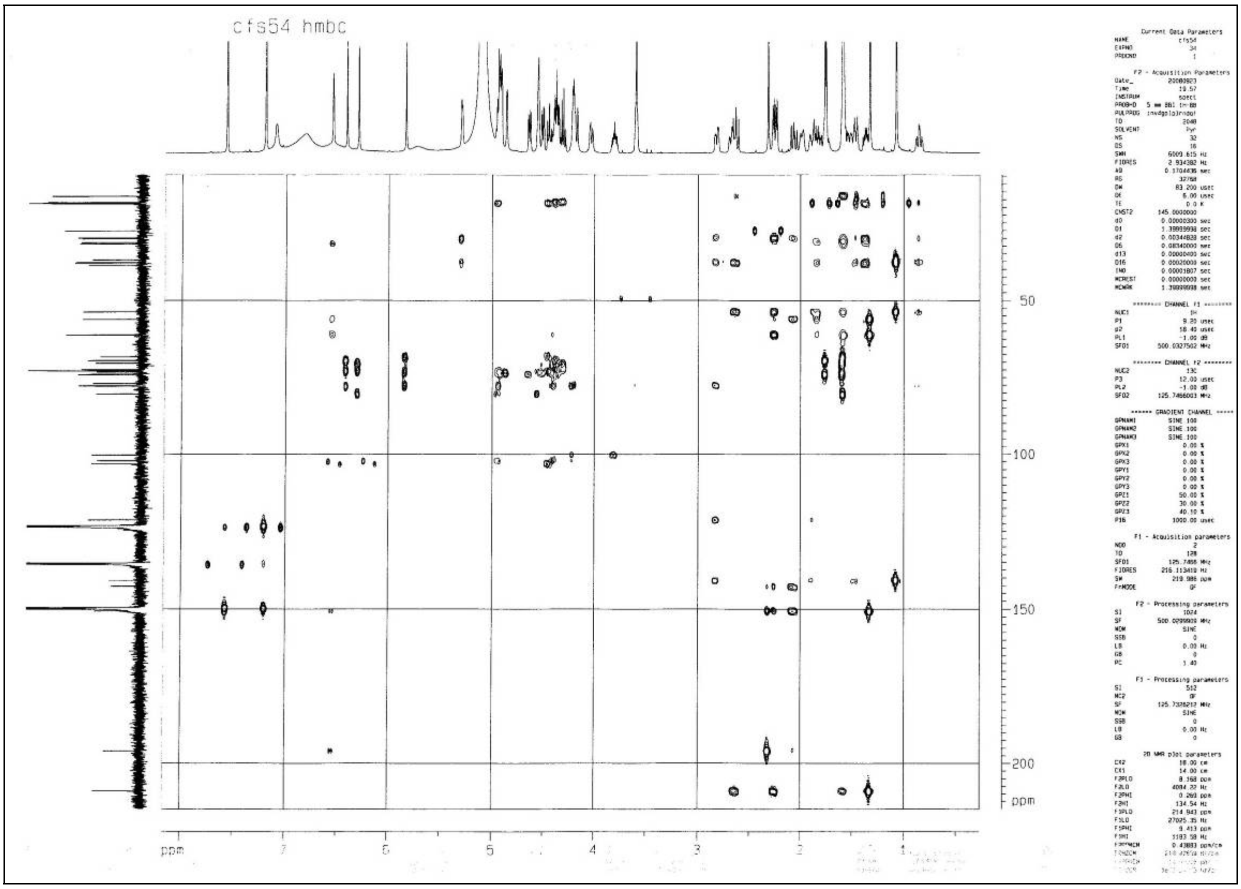

Figure S16. HMBC spectrum of ypsilandroside R (3) in C_5D_5N.

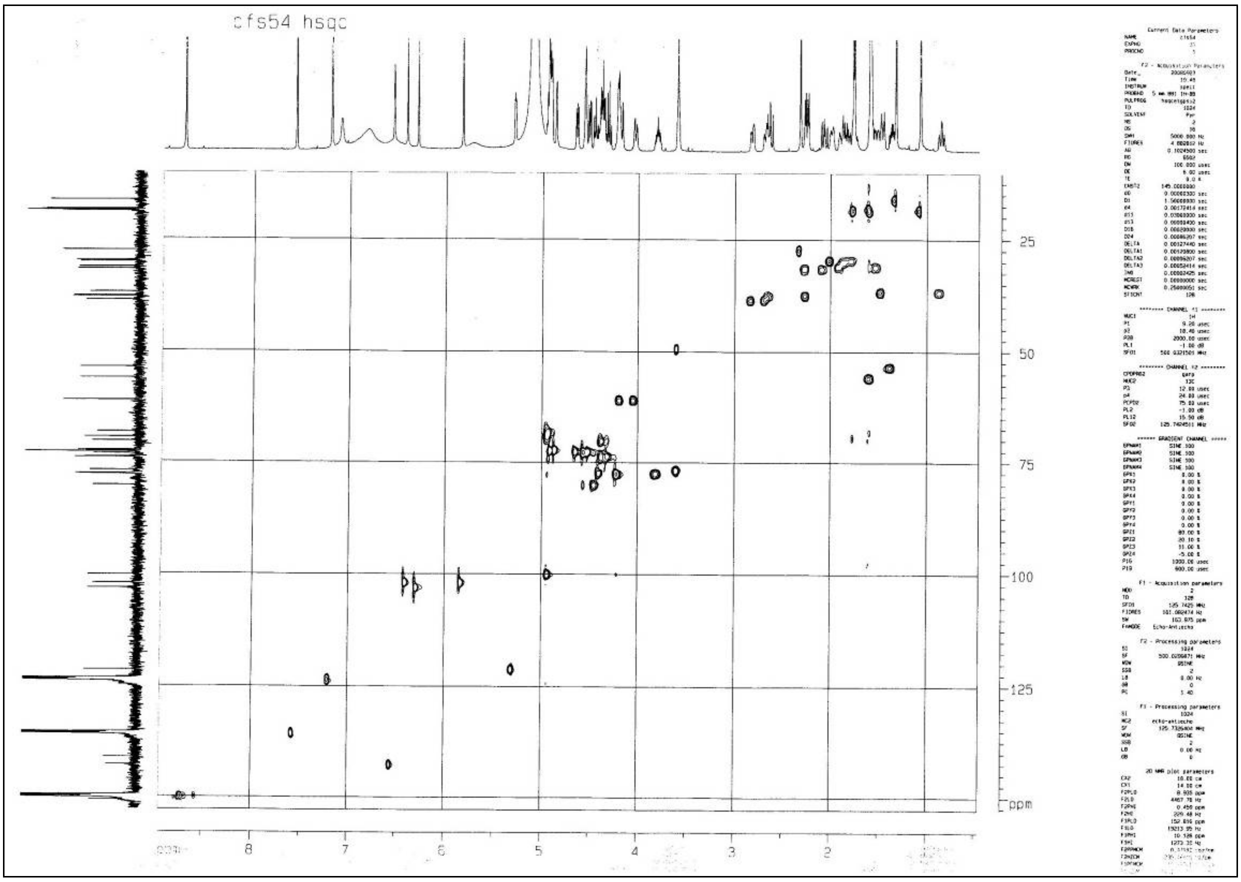

Figure S17. FAB-MS spectrum of ypsilandroside R (3) in C_5D_5N.

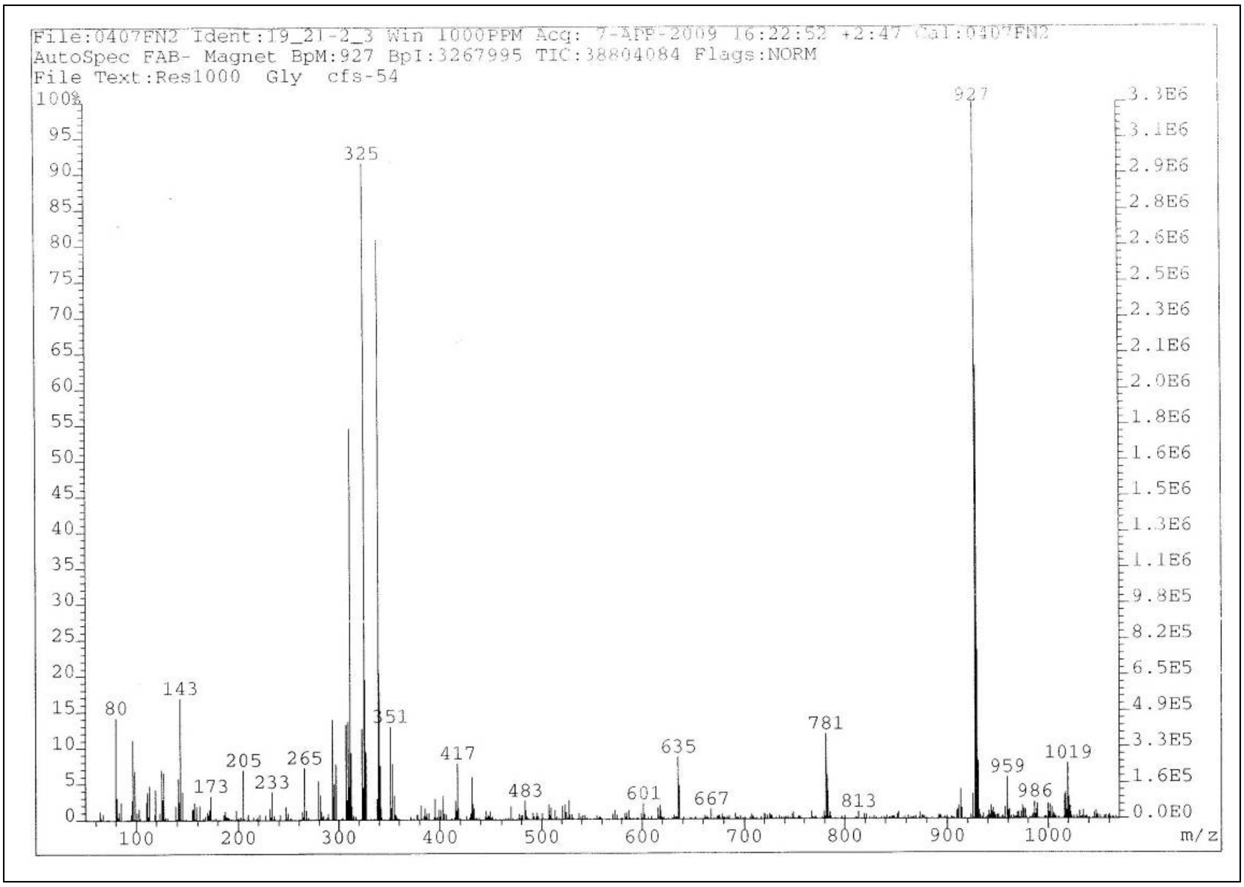

Figure S18. HR-ESI-MS spectrum of ypsilandroside R (3) in C_5D_5N.

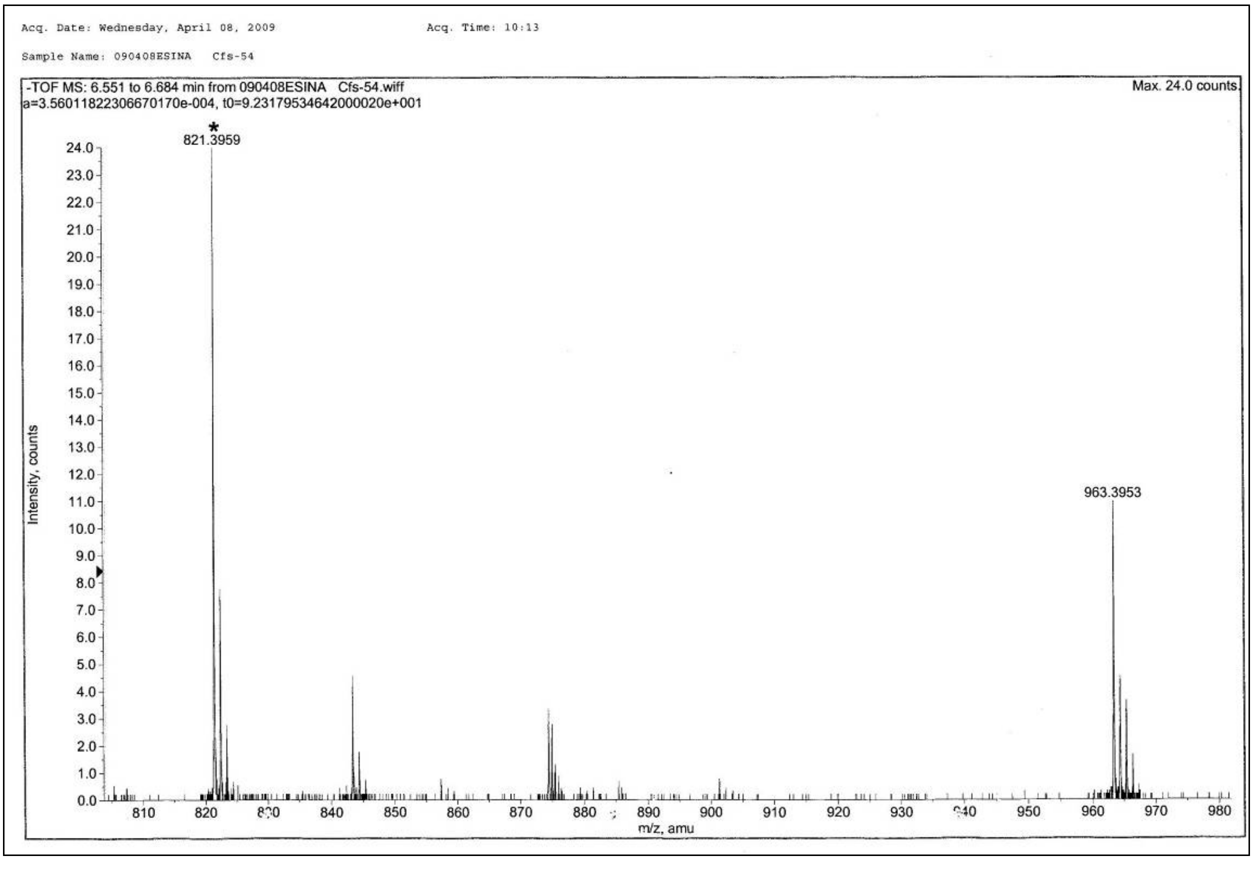

Supplement: Supplementary file 1 — Supplementary material, approximately 1.44 MB. [file 13659_2011_39_MOESM1_ESM.pdf]
